# Rapid Diagnosis of Lung Tumors, a Feasability Study Using Maldi-Tof Mass Spectrometry

**DOI:** 10.1371/journal.pone.0155449

**Published:** 2016-05-26

**Authors:** Geoffrey Brioude, Fabienne Brégeon, Delphine Trousse, Christophe Flaudrops, Véronique Secq, Florence De Dominicis, Eric Chabrières, Xavier-Benoit D’journo, Didier Raoult, Pascal-Alexandre Thomas

**Affiliations:** 1 Service de chirurgie thoracique et des maladies de l'œsophage, Pôle cardio-vasculaire et thoracique, Centre Hospitalo-Universitaire Nord, Assistance publique-Hôpitaux de Marseille, Aix-Marseille université, Marseille, France; 2 Unité de Recherche sur les Maladies Infectieuses et Tropicales Emergentes, URMITE CNRS 7278 IRD 198 INSERM U1905, IHU Méditerranée Infection, Faculté de Médecine, Aix-Marseille Université, Marseille, France; 3 Service d'anatomie pathologique, hôpital Nord, Aix Marseille université, Marseille, France; 4 Pôle des Maladies Infectieuses et Tropicales Clinique et Biologique, Fédération de Bactériologie-Hygiène-Virologie,Centre Hospitalo-Universitaire Timone, Assistance publique des hôpitaux de Marseille, Marseille, France; 5 Service desExplorations Fonctionnelles Respiratoires Centre Hospitalo-Universitaire Nord, Pôle cardio-vasculaire et thoracique, Assistance Publique Hôpitaux de Marseille, Marseille, France; West German Cancer Center, GERMANY

## Abstract

**Objective:**

Despite recent advances in imaging and core or endoscopic biopsies, a percentage of patients have a major lung resection without diagnosis. We aimed to assess the feasibility of a rapid tissue preparation/analysis to discriminate cancerous from non-cancerous lung tissue.

**Methods:**

Fresh sample preparations were analyzed with the Microflex LT^TM^ MALDI-TOF analyzer. Each main reference spectra (MSP) was consecutively included in a database. After definitive pathological diagnosis, each MSP was labeled as either cancerous or non-cancerous (normal, inflammatory, infectious nodules). A strategy was constructed based on the number of concordant responses of a mass spectrometry scoring algorithm. A 3-step evaluation included an internal and blind validation of a preliminary database (n = 182 reference spectra from the 100 first patients), followed by validation on a whole cohort database (n = 300 reference spectra from 159 patients). Diagnostic performance indicators were calculated.

**Results:**

127 cancerous and 173 non-cancerous samples (144 peripheral biopsies and 29 inflammatory or infectious lesions) were processed within 30 minutes after biopsy sampling. At the most discriminatory level, the samples were correctly classified with a sensitivity, specificity and global accuracy of 92.1%, 97.1% and 95%, respectively.

**Conclusions:**

The feasibility of rapid MALDI-TOF analysis, coupled with a very simple lung preparation procedure, appears promising and should be tested in several surgical settings where rapid on-site evaluation of abnormal tissue is required. In the operating room, it appears promising in case of tumors with an uncertain preoperative diagnosis and should be tested as a complementary approach to frozen-biopsy analysis.

## Introduction

Some lung cancer patients reach the operating room without ever having received a preoperative etiological diagnosis, as many as 46% in a recent analysis [1]. The latest recommendations from expert lung cancer societies include the use of *all available* methods to provide a cancer diagnosis prior to major lung resection [2,3]. At the early stages, when a tumor is small in volume, or is too deep-seated to be reached by fine-needle aspiration or transbronchial biopsy, histological identification prior to parenchymal resection is often lacking. Despite a probability-based diagnostic algorithm for surgical decision-making in patients with undiagnosed nodules [3], a recent study reported that 8% of nodules from those operated on without a preoperative certain diagnosis were benign [1]. Because lung resection can significantly impair lung function, it appears crucial to establish whether a nodule is of cancerous origin or not at the time of surgery, in order to minimize the surgical procedure.

To obtain a tumor diagnosis during surgical resection, histology is traditionally performed on frozen sections, which requires a pathologist in or near the operating room. The precision and the degree of certainty of this examination can be lower than that of definitive histology [4], which is the main pitfall in the event of cancer-mimicking inflammatory and fibrotic lesions [5,6]. Definitive pathological examination has been enriched with the help of identification of specific markers. Conversely, frozen section is a fast and simplified examination which is poorly connected to recent technological advances and which would be greatly enhanced by the use of a complementary, rapidly-performed assay.

Matrix-assisted laser desorption ionization time-of-flight (MALDI-TOF) mass spectrometry (MS) is a proteome profiling method which is rapid, precise and uses minimal biological materials, such as for example 1 mm^3^ for lung tissue [7]. Several attempts to identify cancer markers or to precisely classify cancerous tumors according to a variety of subclasses have led to disappointing results in most cases, even when using complex purification and standardization methods [8,9]. Complex analytical methods and genetic algorithms can be used to generate classification models based on the mass (m/z) obtained from MALDI-TOF MS proteome or lipidome analysis. Using this methodological approach, acceptable probability levels of classification were obtained for lung tumors [7,10–12] and liver tumors [13], particularly when MALDI-TOF analysis was coupled with electronic microscope imaging or previous histological tracking, focusing on pre-selected tumor spots [7,14]. Another approach was to incorporate mass spectrometry analysis directly in the operating room, especially with DESI-MS in brain tumors [14]. To date, there is no simple and fast-track protocol that could complement frozen section analysis in order to rapidly determine whether a sample is cancerous.

The objective of the present study was to assess the feasibility and diagnostic contribution of a proteomic analysis using MALDI-TOF applied to fresh lung tissues after minimal preparation. We hypothesized that rapid MALDI-TOF MS analysis could accurately classify tumoral lung tissue of unknown origin as either cancerous or non-cancerous.

## Materials and Methods

### Patients

Between February, 2013 and February, 2015, pairs of tumor and peripheral lung samples were collected from patients undergoing operations in the thoracic surgery unit at the Hôpital Nord in Marseille (France). Informed consent was obtained for each participant, and the research was approved by the ethics committee of the French thoracic surgery society (CERC-SFCTCV-2012-1-31-11-35-32-DeFl). In patients eligible for the surgery, the suspicion of cancer was graded as ‘certain’ on the basis of a preoperative pathological examination, ‘probable’ on the basis of algorithm guidelines or ‘possible’ when no preoperative diagnosis had been obtained and the algorithm criteria were not met.

### Sample Preparation and Analysis

The main resected specimen was sent for standard and immuno-histochemical analyses to the university hospital pathology laboratory and served to obtain a definitive tumor classification. Pathological examination was performed by specialized pathologists recognized for their technical knowledge in lung cancer [15], and in accordance with the current guidelines. Histological type and pTNM score were thus determined as recommended by WHO standards [16].

**Fig 1:**
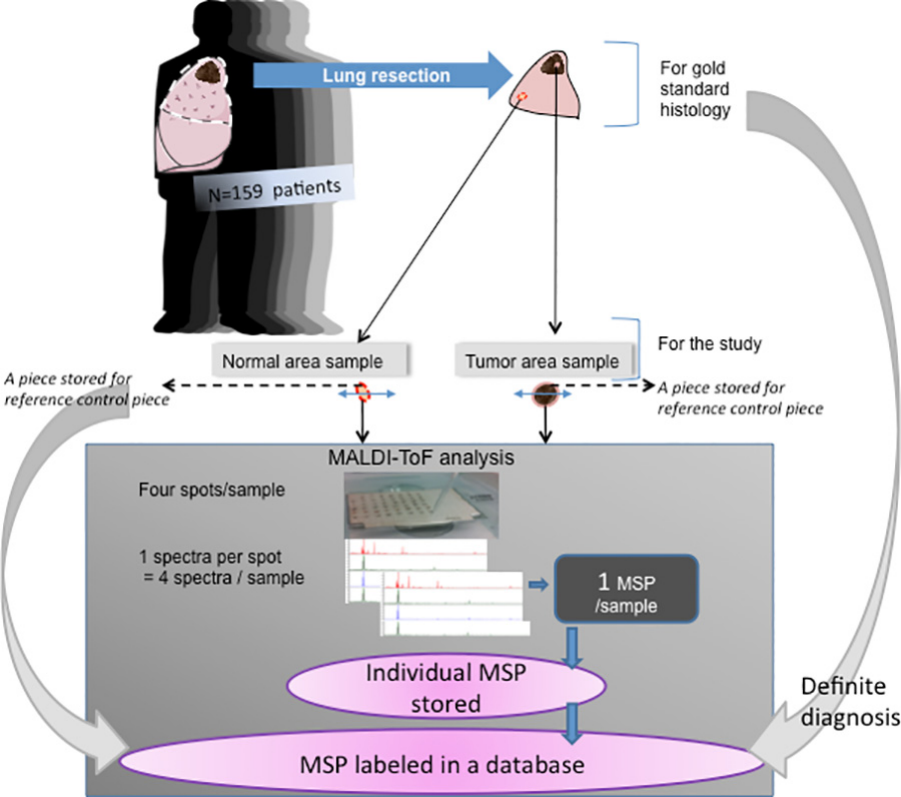
Global design for developing a database after definitive diagnosis was obtained.

During the surgical procedure and immediately following lung resection, one or more biopsies were sampled from the non-tumoral and tumoral areas as guided by the macroscopic appearance. The target size of the biopsies was 1–2 mm^3^.As often as possible, we divided the sample into two equal parts. One fragment was stored in formalin for complementary histological analysis (the complementary reference fragment), while the second was immediately used for the MALDI-TOF MS analysis (Fig 1).

Fragments destined for MALDI-TOF MS analysis were subdivided and weighed (range: 0.01 g to 0.2 g). As previously described [7], sterile water was added to the biopsies to obtain a 10% dilution. After homogenization using IKA ULTRA-TURRAX T25 (IKA^®^-Werke GmbH & CO. KG. Staufen, Germany) at 17,000 rpm for 2 minutes, 100 μl was taken and added to 1.5 ml of sterile water to obtain a 1/160 dilution. Samples of less than 0.01 g were directly diluted in 1.6 ml. Then, 1.5 μl of this solution was applied to a 96 polished steel target. After drying, 1.5 μl of the HCCA α-Cyano-4-hydroxycinnamic acid matrix was added for ionization.

The matrix was prepared daily to obtain a 10 mg/ml of HCCA concentration in a standard solvent (acetonitrile 50%, water 47.5% and trifluoroacetic acid 2.5%). The air-dried targets were measured immediately. Each analysis was performed in quadruplicate and generated four spectra, as adapted from Seng et al.[17] for bacterial identification and as previously validated for human lung samples (international Patent WO2013/150204 A1). In an independent experiment, we tested the reproducibility of the preparation/analysis methods on 20 samples (10 cancerous and 10 non-cancerous) that were subdivided in two pieces and processed independently. Duplicates were not included in the database.

The MALDI-TOF measurements were performed with a MicroflexLT (BrukerDaltonic, Bremen, Germany) mass spectrometer laser. The spectra were recorded in the positive linear mode (delay: 170 ns; ion source 1 (IS1) voltage: 20 kV; ion source 2 (IS2) voltage: 16.65 kV; lens voltage: 7.20 kV; mass range: 2 kDa to 20 kDa). Each spectrum was obtained after 240 shots in the automatic mode for the variable laser power, and the acquisition time ranged from 30 seconds per spot to 1 minute. All signals with a signal-to-noise ratio > 3 in a m/z range of 2000–20 000 Da were automatically acquired using the AutoXecute acquisition control in the FlexControl software®. The spectra of the four spots for each tissue mix were imported into the BioTyper-RTC ^TM^ version 3.0 software (BrukerDaltonik GmbH). The calibration of the MS was fully automated and performed with a commercial solution (BTS: Bacterial standard test) and the procedure was completely automatic (Biotyper RTC user manual)

**Fig 2:**
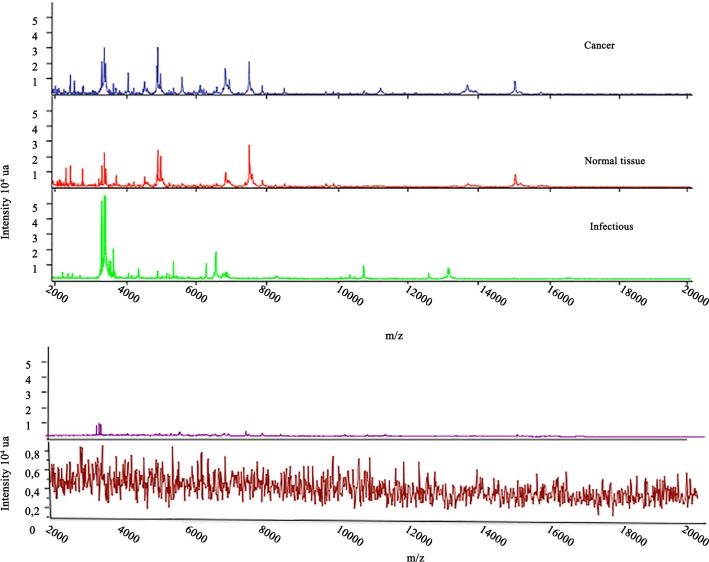
Top: representative spectra of each subclass: Cancerous, Non-cancerous (infection) and peripheral lung (good quality spectra). Bottom: example of two visual template of poor quality spectra (in same scale and in zoom scale).

For each sample analysis, the target was simultaneously tested with an inactivated strain of E. coli (objective score > 2.1) as the positive control and with an HCCA-only matrix as the negative control (objective score < 1.5). All spectra were controlled using the Flexanalysis® v3.4 software (Bruker Daltonic, Bremen, Germany). We checked the quality criteria of the spectrum for global aspect and intensity: intensity above 10^4^, horizontal baseline curve and presence of visually identifiable peaks. An example of high quality and defective spectra is shown in Fig 2.

The subsequent analysis was performed using the MALDI-Biotyper® software (BrukerDaltonic, Bremen, Germany). This software uses spectra identification by searching for homologies with the reference main spectra, named MSP, stored in a custom database. An MSP is obtained from all deposits of a single sample during the same experiment. After comparison of the new spectrum with the database, a comparison score was produced (range 0–3) against each stored MSP. According to the manufacturer’s recommendations for spectra classification (commonly used for bacteria spectra) and recently validated for human lung spectra in the international patent reference WO2013/150204 A1, a score above 1.9 signifies a very high probability of homology between a new spectra and an MSP from the database, while a score below 1.7 signifies an uncertain homology. The results are presented hierarchically from the highest to lowest scores. At the time of data acquisition, each spectrum was numbered anonymously.

When the final pathological diagnosis was obtained, the corresponding MSPs were retrospectively tagged C (cancerous) or NC (non-cancerous).

The cancer class was defined as the group of spectra from primary lung cancer or metastasis.

The non-cancerclass corresponded to the spectra from the peripheral samples as well as those from the non-malignant disease samples. Each cancerous and non-cancerous sample was tested in triplicate.

### Sample Classification Strategy

We designed a sample classification strategy and tested its diagnostic performance in 3 steps. This classification strategy was to take into account the first two best-fit MALDI-Biotyper® comparison scores (> 1.9) among all the comparison scores listed and to attribute the sample to a definitive class depending on the n/8 concordant answers observed. From the eight answers per sample, we could thus obtain from none to eight concordant answers, i.e., matching with different MSP of the cancer class. The minimum number of concordant answers was tested to assess the best diagnostic strategy. When no MSP was found with a comparison score > 1.9, the analysis was considered to be non-contributive and the final response was given as ‘unknown’.

### Assessment of the Classification Strategy Diagnostic Performances

In the first step, the diagnostic performance of a strategy was tested on a preliminary database created with spectra from the first hundred patients (including both cancerous and non-cancerous samples). In the second step, the diagnostic performance of the strategies was assessed by analyzing blindly the samples from the next 59 patients (external validation). The third step aimed to test the influence of the database’s size on the diagnostic performance of our classification strategy and used a definitive database including the MSP from the entire cohort of 159 patients.

**Fig 3:**
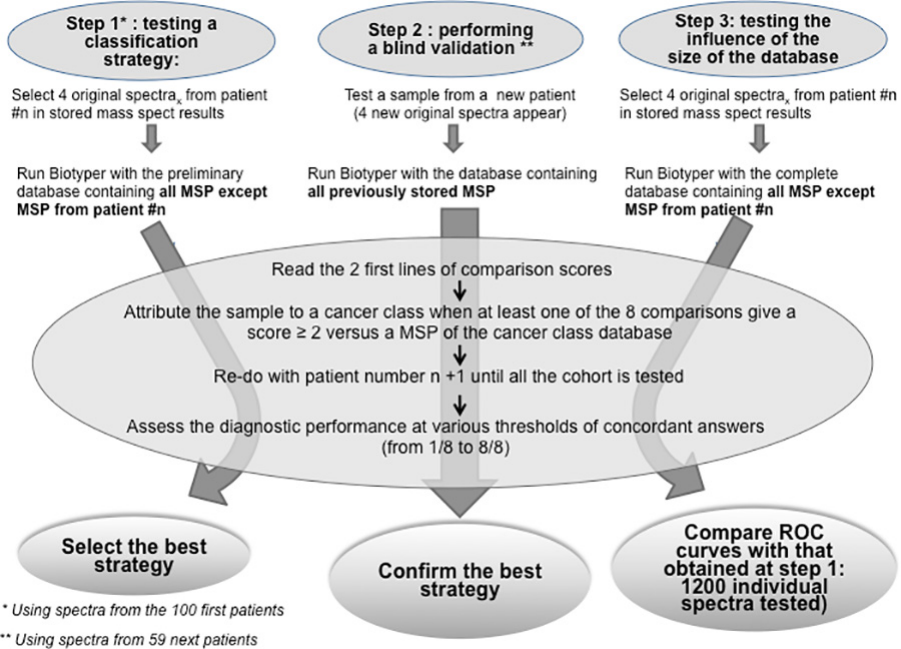
Summary of the 3 evaluation steps of our classification strategy.

At the time of diagnostic performance testing in step 1 and step 2, for each sample to test, the corresponding stored MSP was removed from the database to ensure heterologous comparison. Finally, for each of the four spectra of a sample, thus unknown from the system, MALDI-Biotyper® software delivered the complete list of comparison scores versus all the MSP present in the data base (i.e., the MSP from the remaining 99 patients at step 1 and from the remaining 158 patients at step 3) and edited hierarchically from the best to the worst fit. Summary is shown as Fig 3.

### Statistical Analysis

Data analysis was performed using the IBM^®^ SPSS^®^ statistics software. Student’s t-test or the Mann-Whitney rank-sum test were used for intergroup comparisons of the general data. The sensitivity, specificity, positive predictive value, negative predictive value and global accuracy were calculated using standard formulae.

Diagnostic performance was evaluated and graphically represented by Receiver Operative Curves (ROCs). Areas under the ROC curves were compared using the DeLong method [18]. A p value ≤ 0.05 was considered to be statistically significant.

## Results

### Patients and Samples

During the two-year period, 159 patients requiring surgery consented to the research and were included. Of these patients, we prospectively processed 303 samples as pairs of tumoral and non-tumoral samples from each patient in 144 cases, but 15 patients had only the abnormal tissue sample, given the small lateral margin of the wedge resection.

**Fig 4:**
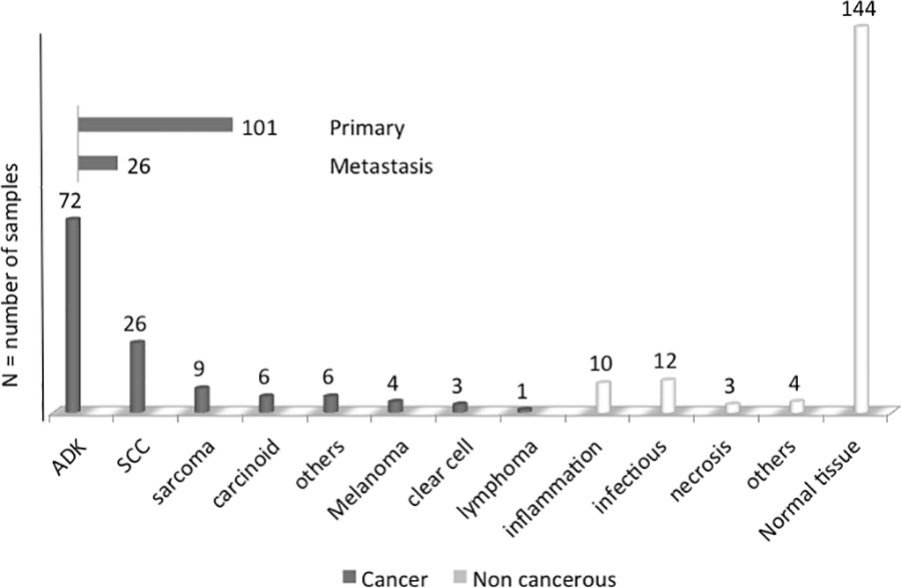
Tissue distribution in the two groups according to the final pathological examination.

**Fig 5:**
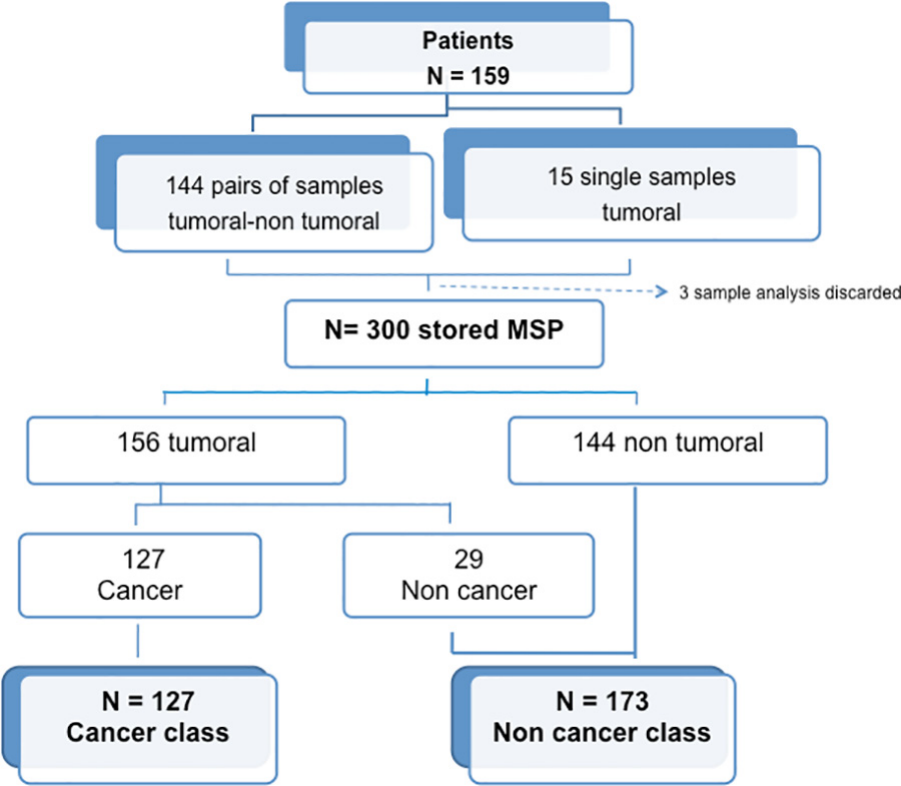
Flow chart for classification of samples and for MSP class allocation.

In three cases, occurring among the first 100 operated patients, the sampling dedicated to the MS analysis was supposed to correspond to a cancerous tissue area, but definitive histological analysis of the study reference control specimen showed only necrotized or nonspecific inflamed tissue, and contrasted with the definitive diagnosis of cancer obtained from the main resected specimen. These 3 samples were excluded from the final analysis, thus the MSP of 300 samples served to assess the diagnostic performance evaluation of MALDI-TOF analysis. According to the definitive pathological diagnoses, 127 tumoral samples corresponded to malignant disease and 29 to non-cancerous nodules; thus, we finally obtained 127 cancerous and 173 non-cancerous samples. Fig 4 shows details for the definitive diagnosis and Fig 5 shows the flow chart for classification of samples and for MSP class allocation). The patients’ demographics, preoperative probability of cancer and the definitive histological diagnoses of resected tumors are reported in Table 1.

**Table 1:** A comparison of the population characteristics between the origins of the cancerous and non-cancerous resected tumors, mean (± standard deviation), median [limits] or number of subjects.

	Cancer group n = 127	Non-Cancer group n = 29	p
**Mean age, years**	61.84 (11.15)	57.75 (11.87)	0.107
**Gender**			0.697
Male, n	72	17	
Female, n	55	12	
**Tobacco** (pack years) Median	35 [0–120]	30 [0–110]	0.299
**Sample weight** (g)			
Mean	0.076 (0.11)	0.072 (0.093)	0.851
**Pre-operative suspicion of cancer** (n)			0.017
Certain	56	8	
Probable	57	10	
Possible	14	10	

The mean weight of the samples used for the MALDI-TOF MS analysis was 0.045 [0.01–0.4] grams for the cancerous samples and 0.045 [0.01–0.23] grams for non-malignant disease (p = 0.851).

### Diagnostic Performance

The median time from resected specimen samplings to MALDI-TOF analysis was 20 +/- 3.2 minutes and corresponded to the pathologist’s response time on frozen section when that occurred.

When the preliminary database was completed (n = 182 MSP from the 100 first patients), spectra from the 20 samples prepared in duplicate were tested for reproducibility: the reproducibility of the preparation/analysis procedure showed that 100% of the 20 samples were classified in the same group as its duplicate and obtained in all cases 7/8 to 8/8 concordant answers with the Biotyper comparison score. The median identification score was 2.42 (minimum 2.06 –maximum 2.68).

#### Step 1: Using the preliminary database to test several classification strategies

Assessing spectra classification for the first 100 patients against the preliminary database enabled defining a best strategy at the threshold for concordant answers of 5/8: i.e., when at least five of the eight Biotyper answers actually matched with a cancer spectra of the preliminary database. This classification strategy showed that a cancerous and a non-cancerous sample were accurately classified with 91.1% sensitivity and 76.2% specificity compared to the definitive diagnosis.

#### Step 2: Blinded test with the preliminary database

The 118 blinded samples from the 59 additional patients were best classified with the 5/8 threshold strategy with a sensitivity of 95.8% and a specificity of 92.9% (Table 2).

**Table 2:** External validation (database from the 100 first patients, n = 118 samples from the 59 following patients blindly tested).

Threshold	TP	FN	TN	FP	Se %	Sp %	Accuracy %
4/8	47	1	64	6	97.9	91.4	94.1
5/8	46	2	65	5	95.8	92.9	94.1
6/8	38	10	66	4	79.2	94.3	88.1

TP: true positives, FN: false negatives, TN: true negatives, FP: false positives, Se: sensitivity, Sp: specificity

#### Step 3: Testing the influence of the database’s size on diagnostic performance

**Fig 6:**
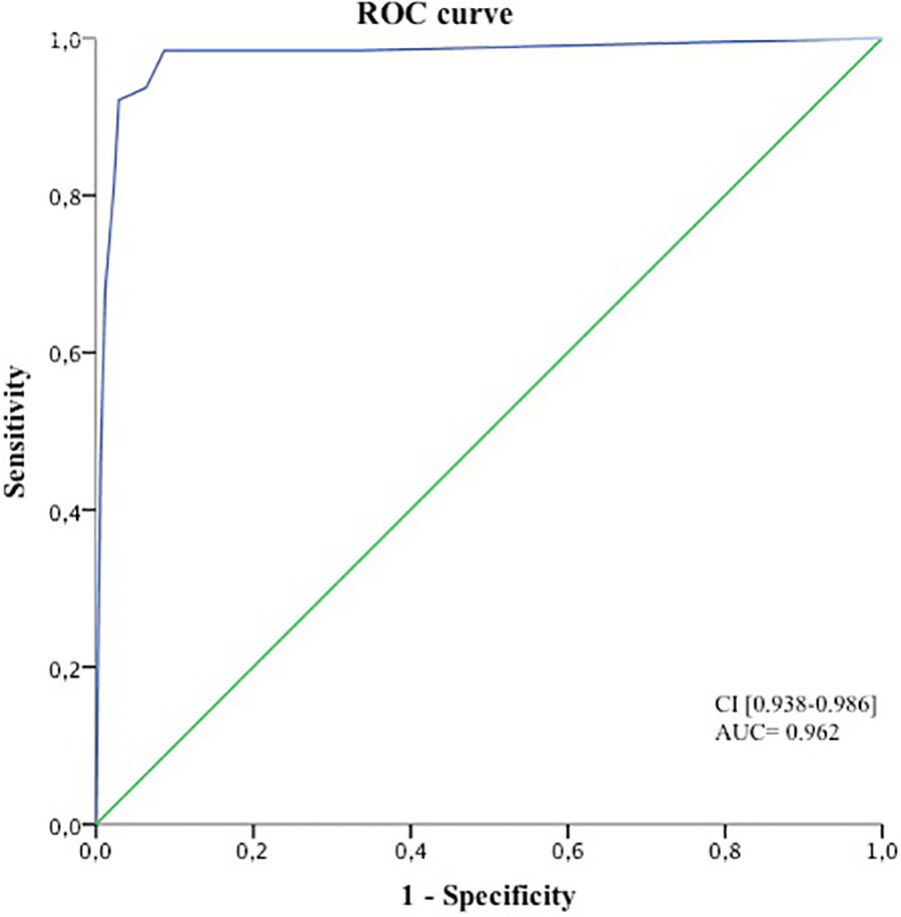
ROC representation for the diagnosis of cancer in the whole cohort, including the tumor and non-tumor tissue samples.

When the preliminary database was questioned for the spectra recognition of the whole cohort samples, the best diagnostic performance of our classification strategy for correctly classifying a cancer sample was once again observed with a threshold of 5/8 concordant answers. This classification method showed 91.3% sensitivity and 94.8% specificity compared to the definitive diagnosis. The same conclusion was obtained when the definitive database (n = 300 MSP) was used, giving the highest performance with a threshold for concordant answers of 5/8 with 92.1% sensitivity, 97.1% specificity and 95% global accuracy (Table 3). The area under the ROC with the definitive database was 0.962, with a 95% confidence interval of between 0.938 and 0.986 (Fig 6). Comparison between the ROCs obtained for the preliminary and the definitive databases did not show significant differences (not shown).

**Table 3:** Diagnostic performance of cancer in the definitive database.

Threshold	TP	FN	TN	FP	Se %	Sp %	Accuracy %
**All samples, n = 300**							
4/8	119	8	162	11	93.7	93.6	93.6
5/8	117	10	168	5	92.1	97.1	95
6/8	104	23	169	4	81.9	97.7	91
**Abnormal tissue only, n = 156**							
4/8	119	8	19	10	93.7	65.5	88.4
5/8	117	10	24	5	92.1	82.8	90.3
6/8	104	23	25	4	81.9	86.2	82.6

TP: true positives, FN: false negatives, TN: true negatives, FP: false positives, Se: sensitivity, Sp: specificity

**Fig 7:**
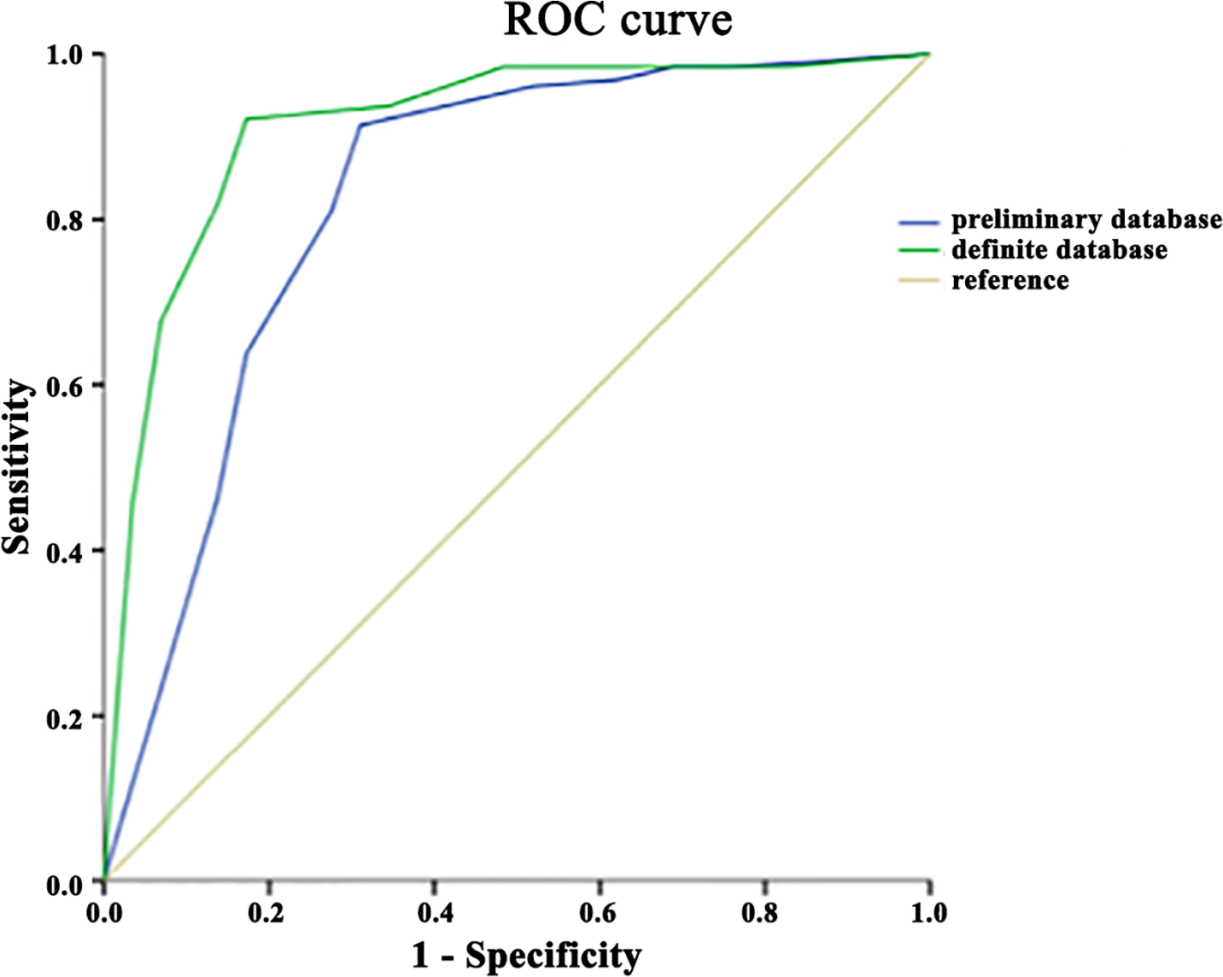
Comparison of the two ROC analyses for the subgroup of tumoral samples, to determine the usefulness of increasing the database.

When only the tumoral samples were considered (127 cancer samples and 29 non-cancerous nodules), the 5/8 threshold was still the most discriminating for diagnosing malignant and non-malignant origins. With the preliminary database, sensitivity and specificity were 91.3% and 69% respectively, and global accuracy was 83.1% (Table 3). Using the definitive database, sensitivity and specificity at the 5/8 threshold reached 92.1% and 82.8% respectively and global accuracy was 90.3%. (not shown) Comparison between the two ROCs (against the preliminary and definitive complete database) showed a greater area under the curve for the tumoral sample classification in the malignant and non-malignant samples, but this was not statistically significant (p = 0.052; Fig 7).

In the subgroup of samples collected from patients with 'possible cancer' based on preoperative probability diagnosis (no preoperative pathological documentation), the 5/8 threshold allowed for a correct diagnosis of cancer with 100% sensitivity, 80% specificity and a global accuracy of 91.6%.

## Discussion

This work represents a continuation of our unit’s research aiming to classify complex pulmonary tissues using MALDI-TOF proteomic analysis [7]. Here, we developed a simple and quick pre-processing method, expecting that if accurate results could reliably be produced, this method could be assessed as a complementary tool in future clinical settings.

The reliability and reproducibility of our pre-processing sample tissue preparation were previously tested on 290 frozen lung tissues [7]. The reproducibility on fresh samples was confirmed here by comparing two of the four spectra with two others from the same preparation (strong homology with Biotyper score > 2 observed throughout the first 480 spectra) as well as by comparing two different specimens from the same 20 test biopsies that were processed and analyzed separately.

This method of homology-based spectra and sample classification produced good discrimination between cancerous tissues and other non-cancerous nodules. The present work focusing on cancerous and non-cancerous pulmonary lesions and rapid MALDI-TOF MS analysis performed in the operating room is of significant clinical relevance.

Whatever the nature of the sample, the diagnosis of cancerous or non-cancerous tissue was obtained with 92.1% sensitivity and 97.1% specificity. When only tumoral tissues were tested, which is the situation most relevant to surgeons, specificity was still good but decreased from 97.1% to 82.8%, meaning that a benign tumoral nodule was sometimes incorrectly diagnosed as cancerous. Although decreased, this diagnostic performance for tumoral tissues is however higher than those published using more sophisticated methods and contrasts with the lack of precise pre-analysis checks for the area of interest. The sampled area was guided only by the surgeon’s visual examination. Lee et al. were able to distinguish adenocarcinoma from squamous cell carcinoma with 84% sensitivity and 77% specificity using a genetic algorithm strategy after histology-directed MALDI-MS [11]. Note that our cohort included several varieties of tumors with more than 15 different histological types of cancerous and non-cancerous nodules.

Data on MALDI-TOF MS applied to complex tissue samples are scarce. Previous work often refers to sophisticated approaches coupling mass spectrometry and optic microscopy, such as MALDI-Imaging [19] or desorption/ionization (DESI-MS) guided by magnetic resonance imaging applied to the lipidome [14]. DESI-MS has been tested in neurosurgery [14]. One of the advantages of combining spectrometry with imagery is the certainty that the spectra will correspond to abnormal cells. In addition, it has a low impact upon the tissue sample. However, tissue imaging coupled with MS requires the presence of a pathologist and implies a large tumor surface for analysis, unsuitable for needle aspiration biopsy, or very small nodules. Moreover, for MALDI-Imaging, the time to obtain an answer takes often several hours[20,21], which appears incompatible with a real-time analysis. Here, the ability of the process analysis to analyze samples as small as 0.01 g gives hope that successful results could be obtained from endo-bronchial ultrasound (EBUS) analysis or computed tomography-guided biopsies.

As a classification strategy, most authors have used the genetic algorithm approach with promising results [7,11–13]. For example, Brégeon et al. analyzed frozen lung sections and correctly discriminated lung cancer from non-tumoral tissue with a sensitivity and specificity of 86.7% and 95.1%, respectively [7]. However, genetic algorithms require a mathematical model established on a trained cohort of spectra before it is validated on the study cohort. The present work was designed differently, based on homology between the spectra generated from a single sample and the complete mean reference spectra (MSP) database divided into two classes.

In bacteriology, the performance of MALDI-TOF MS analysis can be easily understood because the material is derived from a clonal population of microorganisms at a concentration higher than 106 [22].In areas other than bacteria, and especially using complex tissue samples, MALDI-TOF MS analysis followed by Biotyper score comparison with a stored reference spectra has been used with success for identifying the origin of meat product species (Flaudrops C and Chabrière E, personal communication) or for distinguishing different arthropod species [23]. Here, we obtained a good diagnostic performance from a complex tissue sample without purification or cell separation. In addition, we found that increasing the reference spectra population by 50% increased sensitivity and specificity from 91.3% and 69% to 92.1% and 82.8% respectively, with a p value close to significance (p = 0.052).

Concerning the biomarkers, the methodology we chose here did not aim to assess a list of specific or discriminant peaks, unlike our previously published one. Interestingly, we tested on the present set of spectra our previously published m/z peaks and genetic algorithm [7] and obtained a recognition capability of 97.8% and a cross validation of 78% (not shown). In addition, a novel genetic algorithm was created, based on the same settings as previously described [7]: it obtained a recognition capability of 96.46% and a cross validation of 88.73% (not shown). It should be noted that the novel genetic algorithm contained the m/z peaks 8567.48, 4964.42 and 9959.9, which are similar to those obtained previously on frozen sections [7] and are especially similar to those reported by Rahman et al., and attributed to the lung tumor protein thymosin, ubiquitin and acyl-CoA binding protein[12].

The proteomic profile of a complex tissue sample should be considered as a phenotypic expression resulting from multiple functional and/or structural molecules. All attempts to identify specific biomarkers responsible for each peak or groups of peaks present in a MS spectra are fastidious challenges that would require a specific study.

In the 24 patients with only a ‘possible cancer’ diagnosis from the preoperative assessment (retrospectively, there were 14 cancerous lesions and 10 non-malignant lesions), our strategy had a global diagnostic accuracy of 91.6% for detecting lung cancer. Due to the good diagnostic performance of the preoperative algorithms, the place of MALDI-TOF analysis appears particularly relevant in cases of uncertain preoperative diagnosis.

In summary, real-time MALDI-TOF MS analysis with a simple and rapid pre-processing method may provide a rapid diagnostic answer with an acceptable diagnostic performance. It would be interesting to test its use on lymph node analysis. From our perspective, this approach could be proposed in addition to frozen section pathological examinations to help surgeons decide if a major lung resection is necessary, especially in cases of tumors with uncertain preoperative diagnosis.

## Abbreviations

MALDI-TOF: matrix-assisted laser desorption ionization time-of-flight
HCCA matrix: α-cyano-4-hydroxycinnamic acid matrix
MSP: mean reference spectra
ROC: receiver operative curve
MS: mass spectrometry
C: cancerous
NC: non-cancerous
EBUS: endo-bronchial ultrasound
