# Correction: Rapid Diagnosis of Lung Tumors, a Feasability Study Using Maldi-Tof Mass Spectrometry

**DOI:** 10.1371/journal.pone.0162021

**Published:** 2016-08-25

**Authors:** Geoffrey Brioude, Fabienne Brégeon, Delphine Trousse, Christophe Flaudrops, Véronique Secq, Florence De Dominicis, Eric Chabrière, Xavier-Benoit D’journo, Didier Raoult, Pascal-Alexandre Thomas

The seventh author’s name is spelled incorrectly. The correct name is Eric Chabrière.

## References

[1] Brioude G, Brégeon F, Trousse D, Flaudrops C, Secq V, De Dominicis F, et al (2016) Rapid Diagnosis of Lung Tumors, a Feasability Study Using Maldi-Tof Mass Spectrometry. PLoS ONE 11(5): e0155449 doi: 10.1371/journal.pone.0155449 27228175 10.1371/journal.pone.0155449 PMC4881980

